# Association of preoperative cone biopsy with recurrences after radical hysterectomy

**DOI:** 10.1007/s00404-021-06145-0

**Published:** 2021-07-21

**Authors:** Rüdiger Klapdor, Hermann Hertel, Laura Delebinski, Peter Hillemanns

**Affiliations:** Department of Gynecology and Obstetrics, Hannover Medical School, Carl-Neuberg-Straße 1, 30625 Hanover, Germany

**Keywords:** Cervical cancer, Radical hysterectomy, Cone biopsy, Laparoscopy, Recurrence

## Abstract

**Objective:**

To evaluate association of preoperative cone biopsy with the probability of recurrent disease after radical hysterectomy for cervical cancer.

**Methods:**

This is a retrospective single-center study. Patients with cervical cancer stage IA1 with LVSI to IIA2 and squamous, adenosquamous and adenocarcinoma subtype were included. Patients were analyzed for general characteristics and recurrence-free survival (RFS).

**Results:**

In total, of 480 patients with cervical cancer, 183 patients met the inclusion criteria (117 with laparoscopic and 66 with open surgery). The median tumor diameter was 25.0 mm (range 4.6–70.0 mm) with 66 (36.2%) patients having tumors smaller than 2 cm. During median follow-up of 54.0 months (range 0–166.0 months), the RFS for the laparoscopic cohort was 93.2% and 87.5% at 3 and 4.5 years, and 79.3% for the open cohort after 3 and 4.5 years, respectively. In total, 17 (9.3%) patients developed recurrent disease, 9 (7.3%) after laparoscopic, and 8 (12.1%) after open surgery. No preoperative cone biopsy (OR 9.60, 95% CI 2.14–43.09) as well as tumor diameter > 2 cm (OR 5.39, 95% CI 1.20–24.25) were significantly associated with increased risk for recurrence. In multivariate analysis, only missing preoperative cone biopsy was significantly associated with increased risk for recurrence (OR 5.90, 95% CI 1.11–31.29)

**Conclusion:**

There appears to be a subgroup of patients (preoperative cone biopsy, tumor diameter < 2 cm) with excellent survival and low risk for recurrence after radical hysterectomy which might benefit from the advantages of laparoscopic surgery.

## Introduction

After publication of the LACC study, controversy arose regarding the role of the laparoscopic approach for cervical cancer [1]. Contrary to previous mostly retrospective analyses, the LACC trial demonstrated inferiority of laparoscopic radical hysterectomy over abdominal radical hysterectomy with a difference in disease-free survival of 10.6% at 4.5 years [1–3]. As a prospectively planned randomized controlled trial, it led to an impressive decrease in laparoscopic radical hysterectomies for cervical cancer [4]. Further retrospective analyses followed, some confirming the results of the LACC trial and others showing equivalent survival rates after laparoscopic compared with open radical hysterectomy [5–10]. Non-standardized surgical techniques and surgical inexperience are discussed as main critics of studies which show reduced survival after laparoscopic surgery. Results of various other studies lead to the hypothesis that survival might depend on failure to prevent tumor cell contamination through the use of uterine manipulators, intracorporal colpotomy or lack of vaginal cuff closure [11–15]. First results show that patients with tumors under 2 cm or who underwent preoperative cone biopsy appear to have excellent survival even after laparoscopic surgery. To date, there is limited data about how these factors influence the likelihood of recurrence after laparoscopic radical surgery for cervical cancer and which patients might be still eligible for laparoscopic radical hysterectomy. In this study, we analyzed our large collective of patients who have undergone radical hysterectomy for factors that influence the probability of recurrent disease.

## Methods

This is a retrospective single-center analysis. Patients who underwent radical hysterectomy between August 2006 and April 2020 at the Hannover Medical School were included in this study. Inclusion was restricted to patients with squamous, adenocarcinoma or adenosquamous subtypes and patients with FIGO (International Federation of Gynecology and Obstetrics, 2009) stage IA1 with lymph vascular space invasion (LVSI) to IIA2. Surgery was mainly performed by two surgeons. Patients with metastatic disease at primary diagnosis were excluded. Routine surgery included detection of sentinel lymph nodes, pelvic lymph node dissection, radical hysterectomy (Piver II/III) and during laparoscopy a vaginal colpotomy according to the national guidelines. Uterine manipulators were routinely avoided. The way of surgical approach was up to the surgeons and patients choice. Data were retrieved from the hospital documentation system and the institution’s tumor patient database. Patient characteristics were collected in a database and analyzed using SPSS 26 (IBM Corp., Armonk, NY). Time to recurrence (TTR) was calculated as the time difference in months between surgery and first evidence of recurrent disease. Univariate and multivariate logistic regression analyses were performed to estimate odds ratios (OR) and 95% confidence intervals (95% CI). Recurrence-free survival (RFS) indicates the proportion of patients with no evidence of disease at a designated time point. Kaplan–Meier curves were used to perform univariate survival analyses. The log-rank test was conducted for significance analysis. Fisher’s exact test was conducted to analyze associations between bivariate categorical variables. The study was approved by the local ethics committee (Nr. 8740_BO_K_2019). All and individualized data will be available after publication and anonymization directly from the corresponding author.

## Results

During the study period, 480 patients with cervical cancer were treated. In this study, 183 patients were included according to the inclusion criteria that were treated by laparoscopic (117) or open (66) radical surgery for cervical cancer stage IA1 with LVSI to IIA2. The median age was 52.5 years (range 31.0–87.0 years). The median tumor diameter was 25.0 mm (range 4.6–70.0 mm) with 66 (36.2%) patients having tumors smaller than 2 cm. In the laparoscopic group tumor diameter and FIGO stage were significantly lower than in the open surgery group. Further details of our cohort are depicted in Table 1.

**Table 1:** Patient characteristics

	Laparoscopic	Open	*p* value
	Median (range)		
Age (years)	52.0 (31.0–87.0)	54.0 (33.0–86.0)	0.165
BMI (kg/m^2^)	24.0 (18.0–53.0)	29.0 (19.0–44.0)	0.025
Tumor diameter (mm)	20.0 (4.6–55.0)	35.5 (0.5–70.0)	< 0.001
Depth of infiltration (mm)	6.0 (1.0–34.0)	10.0 (1.8–45.0)	0.006
Follow up (months)	54.0 (0–166.0)	25.5 (0–129.0)	0.038

	*N* (valid %)	Open	*p* value
FIGO stage			
IA2	7 (6.0%)	1 (1.8%)	< 0.001
IB1	97 (82.9%)	32 (58.2%)	
IB2	11 (9.4%)	14 (25.5%)	
IIA1	1 (0.9%)	5 (9.1%)	
IIA2	1 (0.9%)	3 (5.5%)	
Grading			
G1	9 (8.0%)	0 (0%)	0.061
G2	72 (63.7%)	40 (64.5%)	
G3	32 (28.3%)	22 (35.5%)	
Tumor size < 2 cm	55 (49.5%)	11 (16.7%)	< 0.001
LVSI (L1)	36 (30.8%)	24 (36.4%)	0.733
Nodal involvement	18 (15.4%)	13 (19.7%	0.455
Adjuvant therapy	24 (20.5%)	31 (47.0%)	< 0.001
Cone biopsy	50.4%	18.2%	
R0	14 (12.0%)	0 (0.0%)	< 0.001
R1	44 (78.6%)	12 (100%)	
R2	2 (3.4%)	0 (0%)	
Vaginal cuff closure	18 (15.4%)	0 (0%)	
Recurrences	9 (7.3%)	8 (12.1%)	0.547
Local	6 (5.1%)	6 (9.1%)	
Distant	3 (2.6%)	2 (3.0%)	
Time to recurrence	12 (3–151)	29.5 (6–63)	0.747

*BMI* body mass index, *LVSI* lymphvascular space invasion

In total, 17 (9.3%) patients developed recurrent disease, 9 of 117 (7.3%) after laparoscopic surgery and 8 of 66 (12.3%) after open surgery. There was no significant difference in incidence or location of recurrences regarding both surgical approaches. Patients after laparoscopic surgery developed recurrent disease with a median time to recurrence of 12 (3–151) months. Only one of these patients primarily presented with a tumor smaller than 2 cm. She developed paraaortic lymph node metastases more than 12 years after radical hysterectomy.

In univariate regression analysis, no preoperative cone biopsy (OR 9.60, 95% CI 2.14–43.09) as well as tumor diameter > 2 cm (OR 5.39, 95% CI 1.20–24.25) showed the strongest association with risk for recurrent disease as shown in Table 2. According to the Fisher’s exact test, there was a significant association between preoperative cone biopsy and tumor diameter < 2 cm (*p* < 0.01).

**Table 2:** Univariate regression analysis on recurrences

	OR	95% CI	*p* value
No cone biopsy	8.88	1.97–40.07	0.004
Tumor size > 2 cm	5.00	1.11–22.61	0.037
Age (years)	0.97	0.96–1.04	0.848
BMI (kg/m^2^)	0.93	0.82–1.05	0.232
Depth of infiltration (mm)	1.01	0.92–1.11	0.866
G3 vs G2	0.67	0.21–2.23	0.540
Adjuvant therapy	0.69	0.20–2.04	0.448
No LVSI	0.41	0.11–1.49	0.174
Tumor size (mm)	1.039	1.00–1.08	0.032
Lymph node metastasis	1.59	0.34–7.32	0.553
Laparoscopic vs open	0.60	0.22–1.65	0.325
Vaginal cuff closure	1.25	0.26–5.97	0.780

*BMI* body mass index, *LVSI* lymphvascular space invasion

**Fig. 1:**
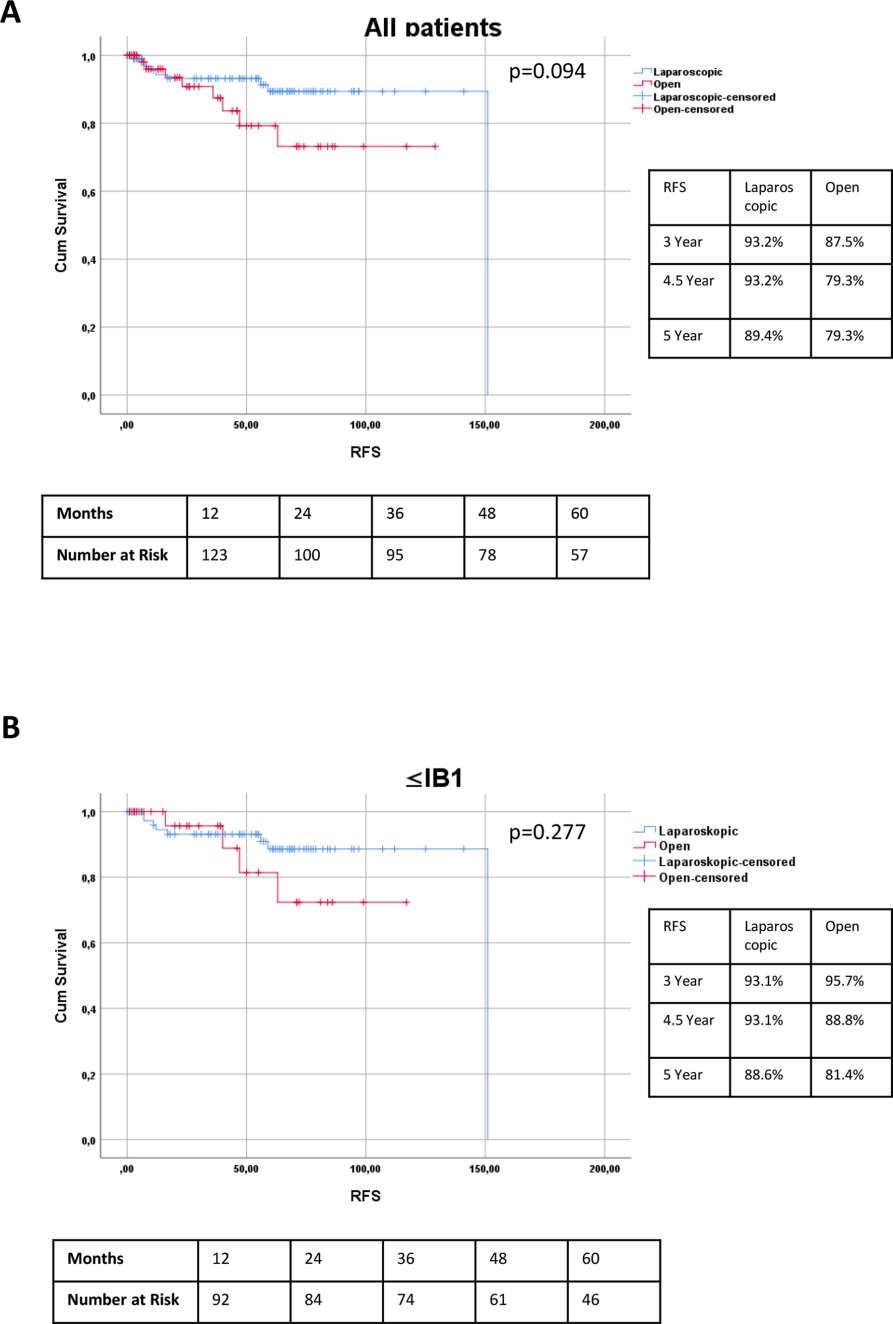
Kaplan–Meier survival curves on RFS for all patients (**A**) and patients according to inclusion criteria of the LACC study (≤ FIGO IB1) (**B**). *TTR* time to recurrence, *RFS* recurrence-free survival

During a median follow-up of 54.0 months (range 0–166.0 months), the RFS for the entire laparoscopic cohort was 93.2%, and for the open cohort 87.5% and 79.3% (laparoscopic vs open log-rank *p* = 0.094) after 3 and 4.5 years as shown in Fig. 1A. Looking only at patients up to FIGO stage IB1 the RFS between both groups was similar resulting in 93.1% (laparoscopic) and 95.7% and 88.8% (open) after 3 and 4.5 years, respectively, as shown in Fig. 1B.

**Fig. 2:**
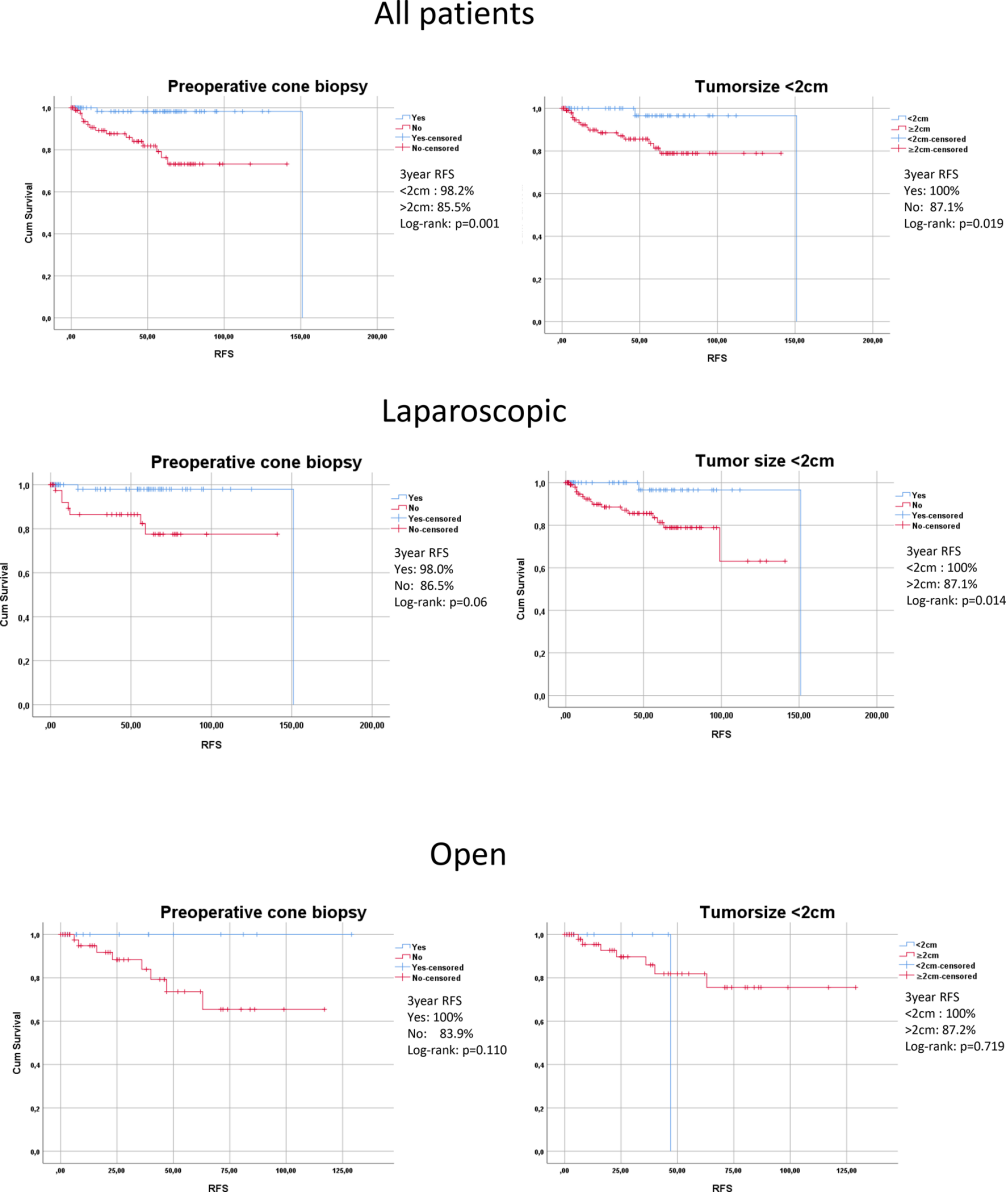
Kaplan–Meier survival curves on RFS for all patients and subgroups after laparoscopic or open radical hysterectomy analyzed for preoperative cone biopsy and tumor size < 2 cm. *TTR* time to recurrence, *RFS* recurrence-free survival

Figure 2 depicts survival curves of all patients, the laparoscopic and open cohort regarding the two strongest risk factors from univariate analysis, preoperative cone biopsy and tumor size > 2 cm. In all patients, there was significantly improved survival for patients with preoperative cone biopsy (*p* = 0.001) and tumor size < 2 cm (*p* = 0.019). Looking at the laparoscopic cohort, patients who underwent cone biopsy before uterine surgery showed improved survival over those patients having no cone biopsy (3-year RFS cone biopsy: 98.0%, no cone biopsy: 86.5%, log-rank *p* = 0.06). There were no patients with completely resected tumor after cone biopsy that developed a recurrence. Similarly, patients with tumors smaller than 2 cm had significantly longer RFS compared to those with larger tumors (3-year RFS < 2 cm 100%, > 2 cm 87.1%, log-rank *p* = 0.014). In the abdominal group, there were no significant differences due to the low number of patients who underwent cone biopsy or presented with small tumors.

In multivariate regression analysis, preoperative cone biopsy was the only factor significantly associated with improved survival. If no cone biopsy was performed, patients had a 5.90 (95% CI 1.11–31.29) increased risk for recurrent disease as shown in Table 3.

**Table 3:** Multivariate regression analysis on probability of recurrences

	OR	95% CI	*p* value
Tumor size > 2 cm	1.32	0.152–11.52	0.800
Laparoscopic vs open	1.63	0.46–5.78	0.451
No cone biopsy	5.90	1.11–31.29	0.037
Tumor size (mm)	1.02	0.96–1.08	0.465

## Discussion

In this analysis of consecutively operated patients with cervical carcinoma FIGO IA1 with LVSI to IIA2, preoperative cone biopsy was the strongest factor associated with reduced risk for recurrence. These data support the theory of the influence of intraoperative tumor spread during radical hysterectomy.

After publication of the LACC study, there was a dramatic change in the treatment of early cervical carcinoma [1, 4, 16, 17]. Even before guidelines recommended abdominal radical hysterectomy as the standard procedure, there was a decline in laparoscopic surgery for early cervical cancer [4]. The LACC trial showed significant inferiority of laparoscopic versus open surgery, with a reduction in disease-free survival from 96.5 to 86.0% after 4.5 years [1]. Similar results were shown in a recent meta-analysis by Nitecki et al., which stated that laparoscopic radical hysterectomy increased the likelihood of recurrence or death by 71% [18]. Unfortunately, they did not evaluate surgical techniques and use of uterine manipulators for their impact on survival. Important results of a retrospective international multicenter study were published by Chiva et al., which showed a disease-free survival at 4.5 years of 79% for laparoscopy and 89% for the abdominal approach [14]. Interestingly, this work demonstrated that the outcomes of laparoscopic surgery were better when no uterine manipulator was used (4.5 years disease-free survival 83% vs. 73%) or a vaginal cuff closure was performed (4.5 years disease-free survival 93% vs. 74%). This correlates with the results of a large patient series published by Köhler et al., in which excellent survival data (4.5 years disease-free survival 95.8%) were achieved for patients undergoing laparoscopic radical hysterectomy with vaginal colpotomy and vaginal cuff closure [11]. A similar approach was suggested by Kanao et al. [19]. Kong et al. hypothesized that intracorporal colpotomy is associated with a threefold decrease in disease-free survival [12]. Although patient and tumor characteristics are not completely comparable between those different studies, these results raise the hypothesis that outcomes of laparoscopic radical hysterectomy depend on surgical technique and the possibility of tumor cell spread into the intraperitoneal cavity [11, 12]. For example, tumor cell spread may occur during intracorporal colpotomy, when intravaginal tumor components have contact with the intraperitoneal cavity, as mechanistically demonstrated by our group [13]. In the present study, after laparoscopic surgery patients with tumors < 2 cm showed only one late recurrence after 12 years which is most likely not associated with possible intraoperative tumor cell spillage since all other local recurrences developed during the first 1.5 years after surgery.

In contrast to the results of the LACC study, which indicated impaired survival for all patients after laparoscopic surgery independently from tumor size, several other analyses comparing laparoscopic with open radical hysterectomy detected comparable results between radical laparoscopic hysterectomy and radical abdominal hysterectomy in patients with tumors < 2 cm [10, 20–24]. In a large analysis of patients treated by laparoscopic or open radical hysterectomy, tumor size > 2 cm was the only factor that characterized patients with increased risk of recurrence by laparoscopic surgery [25]. Of particular interest are the results of the SUCCOR study, in which all patients with preoperative cone biopsy were excluded, which leads to a high-risk patient collective as shown in our study [14]. This analysis reported a comparably low 4.5-year disease-free survival of 79%. Interestingly, the group of patients in which potential tumor cell contamination was attempted to be reduced by protective measures achieved a significantly better 4.5-year disease-free survival of 93%. However, most of the aforementioned studies that suggested a reduced risk for small tumors did not evaluate preoperative cone biopsy.

Interestingly, in our study, of all patients who had received macroscopic tumor resection by preoperative cone biopsy, only one local recurrence was found. There was no recurrence in patients with completely resected tumors by cone biopsy. Preoperative cone biopsy was the only factor significantly associated with reduced risk for recurrences in multivariate analysis with an OR 5.90 (95% CI 1.11–31.29). These results correlate with the study of Casarin et al. in whose analysis a risk reduction of 71% (HR 0.29, 95% CI 0.13–0.91) was shown for patients who received preoperative cone biopsy [23]. Similar results were reported by Uppal et al. [24]. These results raise the question whether there is a causal risk reduction by peroperative cone biopsy. Resection of all macroscopic visual tumor reduces the chances for tumor cell spillage during colpotomy. On the other hand, preoperative cone biopsy, as shown in our study, is associated with smaller tumors which might on the other hand be responsible for improved results. However, multivariate analysis indicates that preoperative cone biopsy is the strongest factor independently associated with risk for recurrence. In our cohort, there were only two patients with residual macroscopic tumor after cone biopsy. Therefore, we cannot conclude whether a macroscopically complete resection is necessary to achieve the optimal results.

In future studies, the role of preoperative cone biopsy to reduce the visible tumor mass should be evaluated especially in laparoscopic surgery. In our cohort, preventive surgical methods to reduce tumor spillage routinely consisted of vaginal colpotomy and non-use of uterine manipulator whereas the formation of a vaginal cuff was more rarely performed. Therefore, it has to be discussed whether in future studies protective surgical methods such as vaginal cuff closure might be omitted in patients with macroscopically resected tumor, since risk for recurrence is extremely low.

This is an exploratory analysis of a retrospective database. Therefore, it can only be used for the generation of hypotheses and the limitations of this analysis must be considered. The aim of this analysis was to assess the recurrences after radical hysterectomy in cervical carcinoma FIGO IA1 with LVSI and above and to evaluate the influence of preoperative cone biopsy believing that selected patients are still be eligible for laparoscopic surgery. We decided not to restrict tumor stage according to LACC inclusion criteria and also include patients with stage up to IIA2. Thereby, we wanted to avoid restricting the cohort too much by retrospective selection. Moreover, additional recurrences provide more data for analysis. The majority of preoperative cone biopsies in our study was performed in FIGO stages IA1-IB1. Due to the low number of recurrences, we cannot determine the effect of preoperative cone biopsy on survival in larger tumors. However, macroscopic tumor residuals after cone biopsy might be associated with worse survival. Since date of preoperative cone biopsy was not documented in most cases, we cannot evaluate the influence of the time interval between cone biopsy and radical hysterectomy. This should be done in already planned prospective studies. More than 90% of all surgeries were performed by two experienced surgeons using standardized surgical technique which allows for a more reliable evaluation on the influence of patient and tumor characteristics compared to multicenter studies. Since there was a selection bias regarding the surgical approach in our cohort, this study was not mainly planned and powered to compare laparoscopic and open surgical approach.

Although the LACC trial did not demonstrate a significant quality-of-life benefit from a laparoscopic procedure, other studies suggest reduced complication rates, shorter hospital stays and lower costs [15, 26–29]. The question arises whether laparoscopic radical hysterectomy is a medically justifiable procedure in certain cases. Our data support the idea that the success of laparoscopic radical hysterectomy depends on the likelihood of intraoperative tumor cell contamination. There appears to be a subgroup of patients (no macroscopic tumor after cone biopsy, tumor diameter < 2 cm) with excellent survival and low risk for recurrence after laparoscopic radical hysterectomy which might still benefit from the advantages of laparoscopic surgery. This analysis supports the initiation of new studies examining laparoscopic radical hysterectomy under conditions that reduce the risk of tumor cell contamination.

## Data Availability

Available on request.
